# Transcriptome analysis of the Japanese eel (*Anguilla japonica*) during larval metamorphosis

**DOI:** 10.1186/s12864-024-10459-z

**Published:** 2024-06-11

**Authors:** Ryusuke Sudo, Taiga Asakura, Takashi Ishikawa, Rui Hatakeyama, Atushi Fujiwara, Komaki Inoue, Keiichi Mochida, Kazuharu Nomura

**Affiliations:** 1 Fisheries Technology Institute, Minamiizu Field Station, Japan Fisheries Research and Education Agency, Minamiizu, Kamo, Shizuoka 415-0156 Japan; 2 Fisheries Resources Institute, Yokohama Field Station, Japan Fisheries Research and Education Agency, Yokohama, Kanagawa 236-8648 Japan; 3 Fisheries Technology Institute, Nansei Field Station, Japan Fisheries Research and Education Agency, Minamiise, Mie 516-0193 Japan; 4 RIKEN Center for Sustainable Resource Science, Tsurumi-Ku, Yokohama, 230-0045 Japan; 5 School of Information and Data Sciences, Nagasaki University, 1-14 Bunkyo-machi, Nagasaki, 852-8521 Japan; 6 Kihara Institute for Biological Research, Yokohama City University, 641-12 Maioka-cho, Totsuka-ku, Yokohama, Kanagawa 244-0813 Japan; 7 RIKEN Baton Zone Program, 1-7-22 Suehiro-cho, Tsurumi-ku, Yokohama, 230-0045 Japan

**Keywords:** *Anguilla japonica*, Leptocephali, Eel genome, Metamorphosis, Transcriptome

## Abstract

**Background:**

Anguillid eels spend their larval period as leptocephalus larvae that have a unique and specialized body form with leaf-like and transparent features, and they undergo drastic metamorphosis to juvenile glass eels. Less is known about the transition of leptocephali to the glass eel stage, because it is difficult to catch the metamorphosing larvae in the open ocean. However, recent advances in rearing techniques for the Japanese eel have made it possible to study the larval metamorphosis of anguillid eels. In the present study, we investigated the dynamics of gene expression during the metamorphosis of Japanese eel leptocephali using RNA sequencing.

**Results:**

During metamorphosis, Japanese eels were classified into 7 developmental stages according to their morphological characteristics, and RNA sequencing was used to collect gene expression data from each stage. A total of 354.8 million clean reads were generated from the body and 365.5 million from the head, after the processing of raw reads. For filtering of genes that characterize developmental stages, a classification model created by a Random Forest algorithm was built. Using the importance of explanatory variables feature obtained from the created model, we identified 46 genes selected in the body and 169 genes selected in the head that were defined as the “most characteristic genes” during eel metamorphosis. Next, network analysis and subsequently gene clustering were conducted using the most characteristic genes and their correlated genes, and then 6 clusters in the body and 5 clusters in the head were constructed. Then, the characteristics of the clusters were revealed by Gene Ontology (GO) enrichment analysis. The expression patterns and GO terms of each stage were consistent with previous observations and experiments during the larval metamorphosis of the Japanese eel.

**Conclusion:**

Genome and transcriptome resources have been generated for metamorphosing Japanese eels. Genes that characterized metamorphosis of the Japanese eel were identified through statistical modeling by a Random Forest algorithm. The functions of these genes were consistent with previous observations and experiments during the metamorphosis of anguillid eels.

**Supplementary Information:**

The online version contains supplementary material available at 10.1186/s12864-024-10459-z.

## Background

Metamorphosis in vertebrates is regarded as being when a developmental stage exhibits remarkable body changes accompanied by a drastic shift in habitat or behavior. A commonly known example metamorphosis is the transformation in amphibians. In amphibians, aquatic larvae (tadpoles) undergo a series of morphological changes including a loss of the tail and hind limbs develop as they transition to terrestrial juveniles. In some teleosts, such as in the Pleuronectiformes (flatfish) and Elopomorpha, they also undergo metamorphosis and transition from a larval stage to immature juveniles.

In flatfish, the asymmetry of body shape appears after metamorphosis [1–3]. In the larval stage, they have symmetrical bodies like most other fishes, and they keep the symmetry and upright swimming position during their pelagic life. Toward the end of the larval stage, the one eye migrates across the top of the head to the contralateral side of the head, resulting in both eyes located on one side of the head, and then the whole-body structure is modified accordingly. Concomitant with these changes, their lifestyle changes from pelagic feeders to sedentary carnivores, lying flat on the bottom of with both eyes facing up. Because flatfish are relatively easy to rear in captivity, researchers have intensively studied the metamorphosis of flatfish mainly from physiology, endocrinology and developmental biology perspectives. It has been shown that the flatfish metamorphosis is primarily controlled by the pituitary-thyroid axis in a comparable way to amphibian metamorphosis [4–9]. These studies have revealed that the developmental changes in various organs are controlled by hormones during metamorphosis [10–15]. Molecular biological studies have also been conducted on the pathway controlling the formation of the left/right axis during early development of flatfish. Recently, transcriptome analysis using next-generation sequencers (NGS) has made it possible to comprehensively understand the genetic dynamics of flatfish during metamorphosis [16–18].

Elopomorphs are composed of the Elopiformes, Albuliformes, and Anguilliformes [19], with the later including marine and freshwater eels. All elopomorphs have leptocephalus larvae, which are a unique larval form with laterally compressed, leaf-like transparent bodies [20, 21] and they undergo a remarkable metamorphosis [22, 23]. During metamorphosis, they transform into a cylindrical form while there is a reduction in both the length and the depth of the body, a loss of teeth, and thickening and pigmentation of the skin [22]. Compared to flatfish, metamorphosis in elopomorphs is not well-documented or studied physiologically. This is because the process occurs mostly in the ocean making it difficult to catch live metamorphosing larvae [24] and rearing methods are not established for any elopomorphs except for the Japanese eel, *Anguilla japonica* [25].

The Japanese eel is a highly valued species for aquaculture. At present, eel aquaculture is totally dependent on wild glass eels captured in estuaries, although the natural stocks of eels have been decreased markedly [26]. Because of this situation, the eel aquaculture industry has recently experienced severe restrictions and rising prices of wild glass eels [27]. To solve this problem, research on the development of artificial glass eel production techniques for Japanese eel has been a long-term effort [25, 28–33]. Over the last two decades, these techniques have been greatly improved [34, 35]. This allowed us to study the mechanism of metamorphosis of leptocephali using larvae reared with using these now well-established rearing methods. Morphological changes during metamorphosis in the laboratory were precisely documented recently [36] and the effects of water temperature, starvation and body size on the onset of metamorphosis have been studied [37]. In addition, it has been suggested that the pituitary-thyroid axis is important in eel metamorphosis, as it is in amphibians and flatfish [38, 39]. However, transcriptome analysis during eel metamorphosis has never been conducted. In the present study, metamorphosizing *A. japonica* leptocephali were subjected to RNA sequencing (RNA-seq) for obtaining basic knowledge about the genetic mechanisms of eel metamorphosis.

## Methods

### Ethics

This project was conducted accordance with the Guidelines for Animal Experimentation of the Fisheries Technology Institute (Japan). All experimental protocols and procedures were approved by Animal Care and Use Committee of the Fisheries Technology Institute (Japan). This study was carried out in compliance with the ARRIVE guidelines.

### Eel larvae

To obtain Japanese eel larvae for analysis, hormonal treatments and artificial maturation of adult eels were conducted in the laboratory. Glass eels purchased from a commercial dealer were feminized by administration of estradiol-17β mixed with commercial eel feed (10 mg/kg feed) for a period of 6 months, because most artificially raised glass eels become males. Male eels purchased form commercial dealer were matured by weekly injection of human chorionic gonadotropin (1000 IU/ kg body weight) as described previously [40]. Semen was cryopreserved in liquid nitrogen until insemination [41]. Female eels were weekly injected with salmon pituitary extract (20 mg/kg body weight) for induction of oocyte maturation. Final maturation was induced by injecting 17α-hydroxyprogesterone (Sigma, St. Louis, MO, USA) at a dose of 2 mg/kg. Eggs were obtained by gently stripping the ovulating female and subsequently fertilizing them with thawed cryopreserved sperm. Larvae hatched from the fertilized eggs were maintained in a 180-L cylindrical polycarbonate tank supplied with filtered seawater at 25 ^o^C until 6 days post hatch (dph). Approximately 1000 larvae were stocked in 20-L acrylic tank supplied with filtered seawater (1 L/min) at 25 ^o^C and then fed 5 times a day at 2 h intervals with a slurry-type diet mainly composed of shark egg, soybean peptide, and krill extract [25]. These larvae were reared up to a maximum of 415 dph. After the larvae had metamorphosed into glass eels, they were then fed blood warms until sampling.

**Fig. 1:**
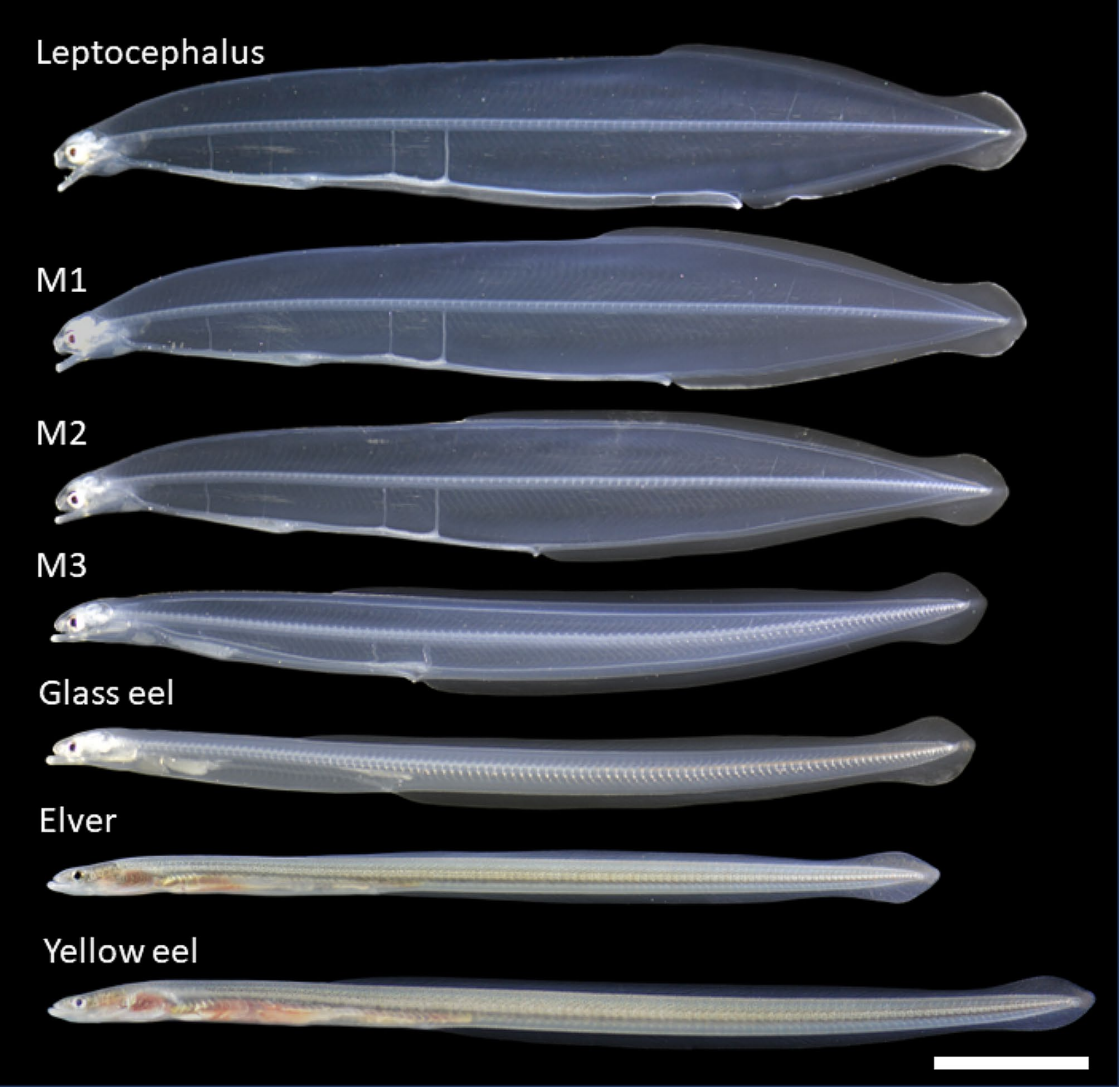
Photographs of the developmental stages of artificially reared Japanese eels (*Anguilla japonica*) from the leptocephalus stage to the yellow eel stage, including 3 metamorphosing stages (M1-M3), the non-feeding glass eel stage and the elver stage when feeding begins

A total 52 specimens were sampled for obtaining larvae that were before, during and after metamorphosis. All specimens were anaesthetized with 400 ppm 2-phenoxyethanol and measured for total length (TL), pre-anal length (PAL), and body depth (BD). The developmental stages during metamorphosis were classified by the morphological indices of proportion, PAL/TL, and BD/TL, and skin coloration as previously described [36, 42] (Fig. 1). Larvae with a PAL/TL ratio ≥ 70% were classified as being in the leptocephalus stage before the onset of metamorphosis. The metamorphic phase, in which drastic body shape change to an eel-like form occurs, was defined to consist of three stages: M1 stage, ≥ 55% and < 70% in PAL/TL and ≥ 10% in BD/TL; M2 stage, ≥ 40% and < 55% in PAL/TL and ≥ 10% in BD/TL; and M3 stage, ≥ 40% and < 45% in PAL/TL and ≥ 7% and < 10% in BD/TL. The glass eel stage was < 40% in PAL/TL and < 7% in BD/TL, and the elver stage was defined by having melanophores on the mediolateral line of the pre-anal body surface. The yellow eel stage has complete guanine deposition on the intraabdominal membrane [42]. After measurement, all were sacrificed by anaesthetizing them for 20 min and were preserved in RNA later solution (Thermo Fisher Scientific, Waltham, MA, USA) until analysis. A total of 28 specimens (4 individuals from each stage) were randomly selected and used as samples for the analysis (Supplementary Table S1).

### Genome annotation

In this study, we performed structural gene annotation of the genome assembly of the Japanese eel (*Anguilla japonica*). As the reference genome sequence, we employed scaffold sequences anchored to genetic markers as previously described for the Japanese eel genome [43]. To comprehensively identify the transcribed structural genes in the species *A. japonica*, we mapped its public Illumina RNA-seq reads and our own Ion Torrent reads to the reference genome using HISAT2 (https://daehwankimlab.github.io/hisat2/) [44] and TMAP (https://github.com/iontorrent/TMAP), respectively. We merged respective BAM files, and subsequently assembled the transcribed regions with StringTie (https://ccb.jhu.edu/software/stringtie/) [45], creating a set of sequences representing the transcribed cDNA. We conducted gene structure annotation using MAKER (https://www.yandell-lab.org/software/maker.html) [46] with the transcribed cDNA and deduced proteome sequences of the genomes of nine fish species (*Astyanax mexicanus*, *Danio rerio*, *Gadus morhua*, *Gasterosteus aculeatus*, *Lepisosteus oculatus*, *Oreochromis niloticus*, *Oryzias latipes*, *Poecilia Formosa*, *Takifugu rubripes*, *Tetraodon nigroviridis*, and *Xiphophorus maculatus*) retrieved from Ensembl (https://asia.ensembl.org/index.html) for evidence-based gene annotation with conserved genes. To identify splicing variants, we mapped the public Illumina RNA-seq reads derived from *A. japonica* to the reference genome using TopHat (https://ccb.jhu.edu/software/tophat/index.shtml) [47] and reconstructed gene structures with Cufflinks (https://github.com/cole-trapnell-lab/cufflinks) [48], which were merged using Cuffmerge. Subsequently, all transcript sequences predicted in the MAKER and RNA-seq reads mapping were evaluated using Transdecoder (https://github.com/TransDecoder/TransDecoder/releases) [49] to identify coding sequences. In this evaluation, the deduced proteome sequences from nine fish species and the set of conserved protein domains of Pfam (http://pfam.xfam.org/) [50] were referenced to assess coding potential. For each genic region, the getorf program of EMBOSS (https://emboss.sourceforge.net/apps/) was used to identify full-length coding sequences, creating models of splicing structure and deducing proteome sequences. Through these steps, we constructed the primary structural gene annotation of the *A. japonica* genome.

Next, to ensure the comprehensiveness of gene expression profiles in the samples collected for this study, we updated the primary genome annotation using both mRNA sequences of *A. japonica* known genes and the metamorphosis RNA-seq data generated in this research. *A. japonica* mRNA sequences including known metamorphosis-related genes, such as thyroid hormone receptors, were retrieved from NCBI. These sequences were processed using CD-HIT (https://sites.google.com/view/cd-hit) [51] for clustering with over 90% similarity, while selecting the longest sequence in clusters as representative sequences. The representative sequences were then mapped to the reference genome using GMAP (https://github.com/juliangehring/GMAP-GSNAP) [52], and the results were integrated with the primary genome annotation. In addition, the metamorphosis RNA-seq reads were mapped using TMAP and assembled on the reference genome using Cufflinks, and the mapping results were further combined using Cuffmerge. We updated the structural annotation by identifying coding sequences with Transdecoder, and splicing structures and proteome sequences using the getorf program. Through these steps, we updated the structural gene annotation of *A. japonica* (Supplementally file 1), providing a reference gene structure that facilitates our metamorphosis RNA-seq analysis.

### RNA-seq analysis

Each sample was dissected and divided into head and body parts. All samples were used in the analysis for the body, while four samples per development stage were analyzed for the head. Total RNA of each part was extracted using the Maxwell RSC simply RNA Kit (Promega, Madison, WI, USA) and mRNA was purified from 3 µg total RNA using the Gene Read Pure mRNA kit (Qiagen, Venlo, Netherlands). Sequencing libraries were constructed from 5 ng of each mRNA sample using the Ion Total RNA-Seq Kit v2 and sequenced with Ion Proton (Life technologies) according to manufacturer’s protocols. The RNA-seq reads were quality checked and trimmed by using CLC Genomics Workbench 9.5.2 software (CLC Bio, Aarhus, Denmark) with default parameters (quality limit = 0.05 and ambiguous limit = 2). Clean reads were aligned with the *A. japonica* reference genome assembly (DDBJ Accession No. BEWY01000001-BEWY01083292) [44] using TMAP program v.3.4.1 using the default parameter settings. The read counts data were generated using the featureCounts tool of the subread package (https://subread.sourceforge.net/) [53] v.1.5, and normalized based on reads per million (RPM).

### Data analysis

A correlation heat map was generated by using Pearson’s correlation coefficient with the standard R (version 4.2.2) function, to compare the differences in gene expression among the developmental stages. These calculations were performed using the logarithm of all gene profiles.

A classification model was created to filter the genes that the characterize developmental stages. For modeling, we adopted the Random Forest (RF) algorithm that used the regression algorithm from scikit-learn, a Python library [54]. RF is an algorithm for classification and regression modeling using an ensemble of decision trees [55] and is frequently employed in recent biomarker discovery and structure prediction studies [56–58]. The filtering of characteristic genes for the developmental stages was performed based on the gene expression profiles using the following procedures [56]. Firstly, a RF algorithm was used to create developmental stage classification models for both the body and head, incorporating all gene profiles. The created models underwent cross-validation using the leave-one-out approach. Next, the feature importance (gini importance) of explanatory variables (genes), obtained from the created models, was used to sequentially add highly ranked genes. The accuracy was then plotted at each iteration of cross-validation. This process was repeated until the accuracy plateaued. The set of genes that reached the plateau were defined as the ‘most characteristic genes’ during metamorphosis.

Correlation network analysis allows for the visualization of overall correlations and the discovery of new relationships [59, 60]. Genes that are highly correlated with the most characteristic genes do not change their cross-validation accuracy rate with variable addition. Therefore, genes with a high correlation (0.9 or higher) with the most characteristic genes were also extracted for network analysis. A network analysis figure was created by connecting the colored nodes that represent most characteristic genes and the black nodes, which are genes that correlate with most characteristic genes with edges. Genes that are correlated with multiple characteristic genes belong to multiple clusters. Plotting of the network analysis was performed with the software Gephi (version 0.10, https://gephi.org/) [61]. To show the increase/decrease of each gene cluster, the expression values were averaged for each cluster after Z-score normalization of RPM values among samples of each gene and are compared for each developmental stage.

Gene ontology (GO) enrichment analysis is a valuable analysis to gain insight into the functions assigned to genes [62, 63]. Based on the clusters created above, we identified functions that respond to the developmental stage changes. We calculated the percentage of genes annotated with a specific GO in each cluster and the percentage of genes annotated with that GO among all genes, and picked up GOs that were observed significantly more frequently. For calculation of significance, the p-value was determined by the Fisher’s exact test (using python library “SciPy” version 1.3.0) and corrected by the Benjamini-Hochberg method. Significant p-values were set at less than 0.05.

## Results

### Overview of transcriptome analysis during metamorphosis

**Fig. 2:**
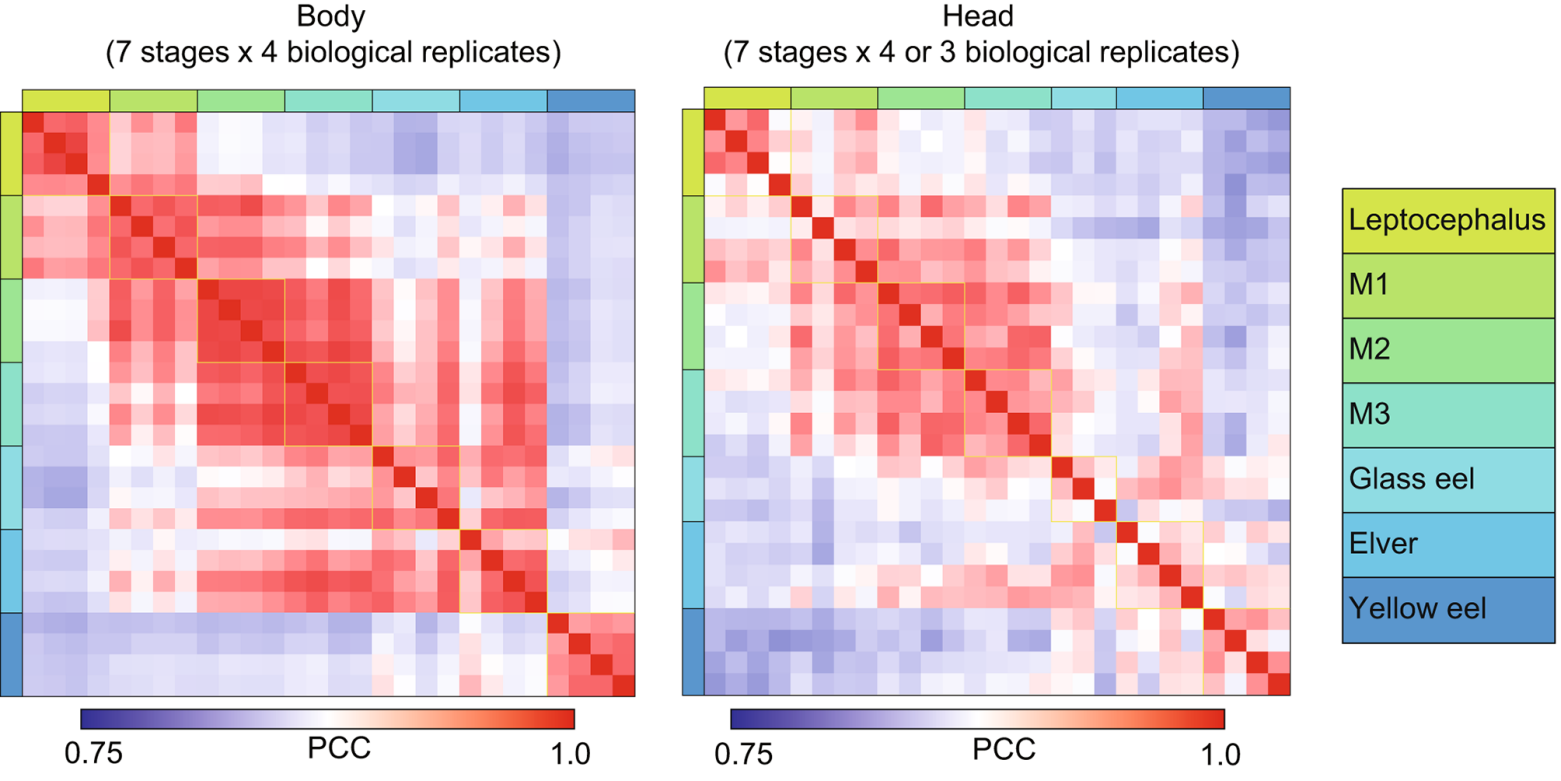
Heat map of Pearson Correlation coefficients (PCC) obtained from the transcriptome datasets based on RPM values for samples from the body (left) and head (right). The PCC values between the samples of the same stages are marked by yellow squares

A total of 354.8 million (Body) and 365.5 million (Head) clean reads from transcriptomic libraries were generated after the processing of raw reads from the different stages of Japanese eels from the leptocephalus to the yellow eel stage. In the head, one of the samples from the glass eel stage was excluded from the analysis because of insufficient data quality. Transcriptome sequencing revealed that more than 96.5% of the total reads in each sample were uniquely mapped reads (Table S2). In both the body and head, higher PCC values in the expression profiles were observed within the same developmental stage (Fig. 2). When compared among stages, the PCC values were relatively high between adjacent stages. We found that the yellow eel stage showed lower PCC values in comparison to all the other stages in the body, showing different expression profiles, but such distinct differences were not observed in the head especially for the glass eel and elver stages.

### Most characteristic genes during metamorphosis

**Fig. 3:**
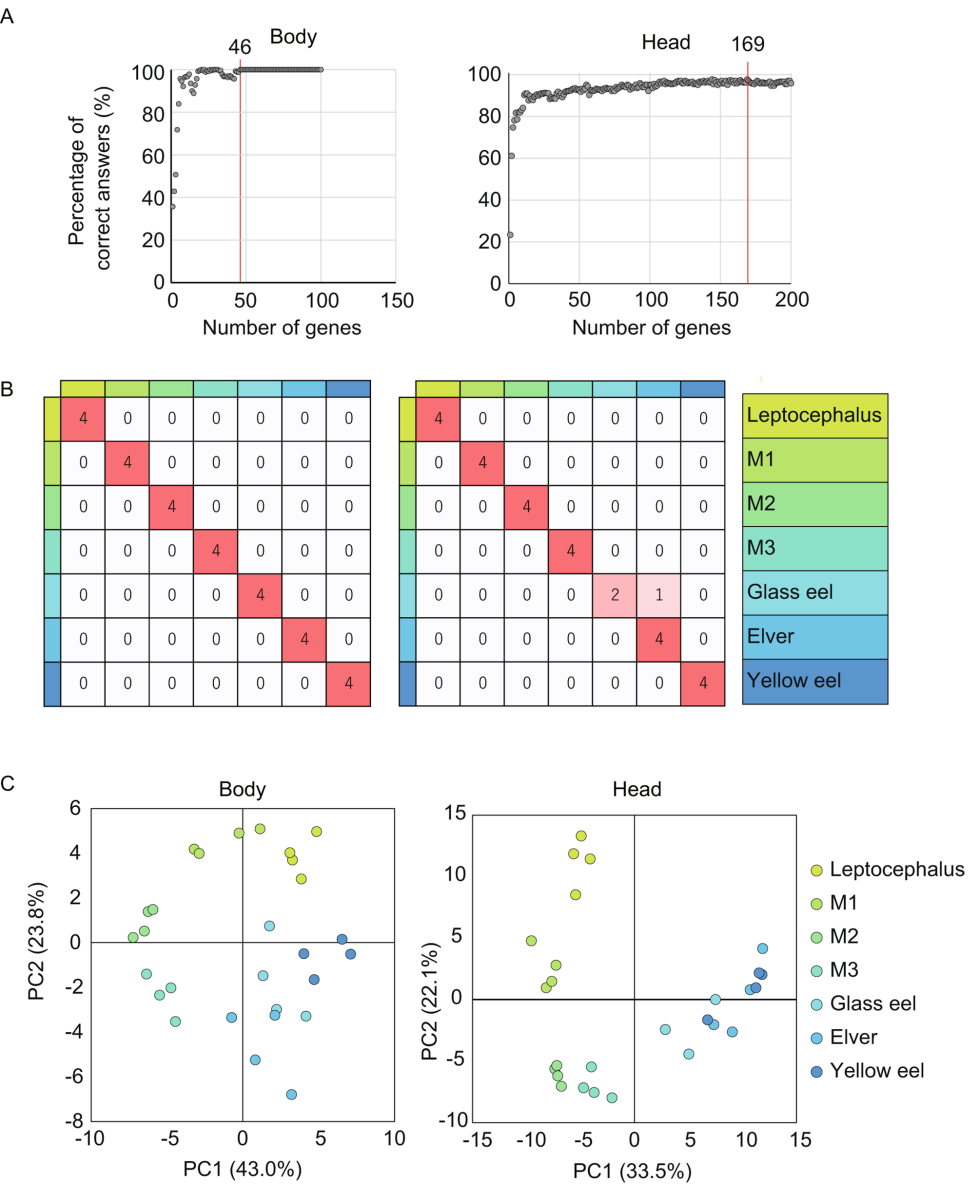
Classification modeling for RNA-seq datasets to filter the genes that characterize the developmental stages. **A**: The feature importance (gini importance) of genes, obtained from each created model. **B**: Cross-validation using the leave-one-out approach. **C**: Principal component analysis of the most characteristic genes during metamorphosis

For validating the classification model created by the RF algorithm, the leave-one-out cross-validation all genes (before filtering the genes) showed correct answer rates of 0.54 and 0.26 for the body and head, respectively. Gene filtering found that body genes were 100% accurate to identify the developmental stages using 46 genes. In the head, 169 genes showed the maximum accuracy (96.3%) and then the accuracy decreased (Fig. 3A, B). From these results, 46 genes selected in the body and 169 genes selected in the head were defined as the “most characteristic genes” (Table 1, Supplementary file 2, 3 and 4). Principal component analysis (PCA) using the most characteristic genes revealed changes in the developmental stages and the characteristics of the increased and decreased expressed genes were captured (Fig. 3C). For the body, the PC1value decreased from M1 to the elver stage, and the PC2 value of the body decreased after the M3 stage (Fig. S1a). In the head, the PC2 value decreased greatly from the leptocephalus stage to the glass eel stage, and the PC1 value increased until the yellow eel stage (Fig. S1b).

**Table 1:** Genes that most often account for the metamorphic phase chosen by the random forest method

Body				Head				
Gene ID	Description	Gene ID	Description	Gene ID	Description	Gene ID	Description	
Angja18g003080	Ankyrin repeat and SAM domain-containing protein 4B	Angja103s000020	Melanopsin-B; AltName: Full=Opsin-4B	Angja7g006600	1-phosphatidylinositol 4,5-bisphosphate phosphodiesterase beta-4	Angja23183s000040	GDNF family receptor alpha-2	
Angja81329s000010	Interferon gamma receptor 1-like	Angja10g004570	---NA---	Angja7g013310	Thyroid hormone receptor beta	Angja2g002530	SPARC	
Angja2g009280	---NA---	Angja11g007790	CD166 antigen homolog A	Angja7g013420	Collagen alpha-1(XXVIII) chain; Flags	Angja2g005070	Succinate dehydrogenase assembly factor 2, mitochondrial	
Angja217s000190	Eukaryotic translation initiation factor 4E-1 A	Angja121s000220	---NA---	Angja7g013770	---NA---	Angja2g007270	Chromobox protein homolog 5	
Angja19g004440	Discoidin domain-containing receptor 2	Angja13g001260	---NA---	Angja7g015920	Annexin A2	Angja2s000050	Cyclic GMP-AMP synthase	
Angja1g009760	Eukaryotic translation initiation factor 3 subunit B	Angja13g004710	Cytoplasmic dynein 1 light intermediate chain 2	Angja7g019950	IgGFc-binding protein	Angja3g001900	Junction-mediating and -regulatory protein	
Angja15g000770	---NA---	Angja14g000590	Grancalcin	Angja7g020070	IGF-like family receptor 1	Angja3g003630	Claudin-5	
Angja4g004690	Rho GTPase-activating protein 18	Angja15g006180	Seipin	Angja81310s000090	Synaptopodin-2	Angja3g007770	C-X-C chemokine receptor type 3	
Angja2g010740	L-lactate dehydrogenase A chain	Angja15g009370	MICOS complex subunit MIC27	Angja101s000190	Semaphorin-3ab	Angja3s000010	Ras-related protein Rab-26	
Angja6g001200	Cytidine deaminase	Angja15g012170	Transmembrane protein 164	Angja103s000040	Glial cell line-derived neurotrophic factor	Angja4088s000010	Immunoglobulin kappa variable 2-29	
Angja85s000070	Glutathione S-transferase Mu 3	Angja178s000040	Green-sensitive opsin-3	Angja10g000090	---NA---	Angja4g003310	Aquaporin-4	
Angja5g010880	Eyes absent homolog 1	Angja178s000070	Protein tyrosine phosphatase domain-containing protein 1	Angja111s000070	Beta-1,3-galactosyltransferase 2	Angja4g004540	Divergent protein kinase domain 1 C	
Angja16g002460	Transitional endoplasmic reticulum ATPase3	Angja23627s000330	Calpastatin	Angja11238s000010	Collagen alpha-1(IX) chain; Flags: Precursor	Angja4g006130	Ankyrin repeat and SOCS box protein 10	
Angja12g003940	Solute carrier family 2, facilitated glucose transporter member 103	Angja2g009160	Electron transfer flavoprotein subunit alpha, mitochondria	Angja11g004870	Pannexin-1	Angja4g006630	Cartilage matrix protein	
Angja7g013710	Alpha-1,3-mannosyl-glycoprotein 2-beta-N-acetylglucosaminyltransferase3	Angja4g008910	Guanine nucleotide-binding protein G(I)/G(S)/G(T) subunit beta-3	Angja12g002150	RanBP-type and C3HC4-type zinc finger-containing protein 1	Angja4g007640	Stromal membrane-associated protein 2	
Angja211s000040	C1GALT1-specific chaperone 1	Angja6g000030	Calcium/calmodulin-dependent protein kinase type 1G	Angja12g007910	1-phosphatidylinositol 4,5-bisphosphate phosphodiesterase eta-2	Angja4g008270	Transcription factor SOX-4	
Angja81314s000020	Anoctamin-5	Angja10909s000020	Collagen alpha-1(V) chain; Flags: Precursor	Angja12g008170	Rho guanine nucleotide exchange factor 19	Angja4g013400	PHD finger protein 1	
Angja5g004160	Divergent protein kinase domain 1 C	Angja7g008480	Transcription factor SOX-11; Short=cSox11	Angja12g008180	---NA---	Angja55803s000010	---NA---	
Angja3s000040	Transcription factor Sox-8	Angja123s000240	---NA---	Angja12g008860	Calcium/calmodulin-dependent protein kinase type 1G	Angja5g001860	Repulsive guidance molecule A	
Angja6g003530	Cerebellin-4	Angja14g000290	Integrin alpha-4; AltName: Full=CD49 antigen-like family member D	Angja131s000310	Growth hormone-regulated TBC protein 1-A	Angja5g007770	Hippocampus abundant transcript 1 protein	
Angja11g007110	Ankyrin repeat and protein kinase domain-containing protein 1	Angja14g000510	Collagen alpha-1(XVIII) chain	Angja13g001090	Epidermal growth factor receptor kinase substrate 8-like protein 2	Angja5g014460	Tetraspanin-13	
Angja81243s000130	Calpain-9	Angja14g000980	Collagen alpha-1(XXVIII) chai	Angja142s000010	Cingulin-like protein 1	Angja5g014490	Serine/threonine-protein phosphatase 1 regulatory subunit 10	
Angja11g002800	TRPM8 channel-associated factor homolog	Angja15g000310	Protein YIF1A	Angja14g004400	Tomoregulin-2	Angja6g001190	Inositol 1,4,5-trisphosphate receptor type 3	
Angja73s000070	Transmembrane 4 L6 family member 4	Angja15g001370	Periostin	Angja14g005420	Gap junction alpha-3 protein	Angja6g010780	---NA---	
Angja12g000630	---NA---	Angja16g006270	Stromelysin-3	Angja14g005790	39 S ribosomal protein L39, mitochondrial	Angja77s000190	Leucine-rich repeat transmembrane neuronal protein 4	
Angja58319s000190	Platelet-derived growth factor receptor beta	Angja19g007100	Translocating chain-associated membrane protein 2	Angja14g006490	Alpha-crystallin A chain	Angja78794s000010	---NA---	
Angja60428s000010	Teashirt homolog 1	Angja3g001120	Collagen alpha-1(II) chain	Angja15g000920	Olfactory receptor 52J3	Angja7g002520	Cyclin-dependent kinase inhibitor 3	
Angja19g005320	Neuroendocrine protein 7B2	Angja5g005940	Paired mesoderm homeobox protein 1	Angja15g003030	Zinc finger HIT domain-containing protein 3	Angja7g004010	Probable sodium-coupled neutral amino acid transporter 6	
Angja1g004530	Protein FAM83F	Angja5g006340	Thioredoxin domain-containing protein 5	Angja15g005750	Ribosomal protein S6 kinase-related protein	Angja7g005460	Latent-transforming growth factor beta-binding protein 2	
Angja3g004990	Zinc finger protein 532	Angja6g005900	Protein snail homolog Sna	Angja15g007100	Migration and invasion-inhibitory protein	Angja7g005470	Protein O-mannosyl-transferase 2	
Angja6g001950	Plexin-A1	Angja7g001020	Thyroid hormone receptor-associated protein 3	Angja15g007310	Serine/threonine-protein kinase PAK 1	Angja7g011530	Mitotic interactor and substrate of PLK1	
Angja14g007880	Krueppel-like factor 5	Angja7g010980	Protein odd-skipped-related 2	Angja15g011540	Poly(U)-specific endoribonuclease-B	Angja7g012080	Dual specificity protein kinase Ttk	
Angja7g006890	Disintegrin and metalloproteinase domain-containing protein 17	Angja7g015200	Peptidyl-prolyl cis-trans isomerase FKBP14	Angja16g001770	Succinyl-CoA	Angja7g015460	Cathepsin K; Flags: Precursor	
Angja28s000100	NADH dehydrogenase [ubiquinone] 1 alpha subcomplex subunit 10, mitochondrial	Angja8g009800	Beta-galactosidase	Angja16g009490	Zinc finger protein 180	Angja7g018350	Hippocalcin-like protein 4	
Angja12g008860	Calcium/calmodulin-dependent protein kinase type 1G	Angja14g003530	Tumor necrosis factor-inducible gene 6 protein	Angja17g000470	Proton myo-inositol cotransporter	Angja7g019410	IgGFc-binding protein	
Angja10g005440	Sulfotransferase 6B1	Angja14g005860	A disintegrin and metalloproteinase with thrombospondin motifs 5	Angja18g001360	Serine/threonine-protein phosphatase PP1-gamma catalytic subunit A	Angja7g019430	LINE-1 reverse transcriptase homolog	
Angja81305s000210	---NA---	Angja2g001240	Alcohol dehydrogenase class-3	Angja18g009770	Hepatic leukemia factor	Angja7g019550	Ribonucleoside-diphosphate reductase subunit M2	
Angja81493s000010	Laminin subunit gamma-1	Angja2g003020	Nephronectin	Angja196s000320	Ankyrin repeat domain-containing protein 29	Angja7g020270	Ankyrin repeat domain-containing protein 9	
Angja81322s000120	RNA-directed DNA polymerase from mobile element jockey	Angja10g005910	Myozenin-1	Angja19g002640	Cation-independent mannose-6-phosphate receptor	Angja80708s000010	T cell receptor beta variable 7-9	
Angja3g000260	---NA---	Angja11g001320	Protein sel-1 homolog 3	Angja19g005110	Syndecan-1	Angja81398s000040	Tripartite motif-containing protein 35	
Angja1g003560	Cdc42 effector protein 1	Angja11s000340	Potassium voltage-gated channel subfamily A member 3	Angja19g005910	---NA---	Angja81493s000010	Laminin subunit gamma-1; Flags: Precursor	
Angja10g004970	Aldose 1-epimerase	Angja12g004840	Kelch domain-containing protein 7 A	Angja19g007310	E3 ubiquitin-protein ligase UHRF1	Angja83241s000010	---NA---	
Angja12g004390	Keratin, type I cytoskeletal 18	Angja13g002970	Electron transfer flavoprotein subunit alpha, mitochondrial	Angja19g007730	Calcium-activated potassium channel subunit beta-3	Angja8g001410	Placenta-specific gene 8 protein	
Angja4g001790	Kelch-like protein 18	Angja149s000060	Myosin-16	Angja19g011140	ER degradation-enhancing alpha-mannosidase-like protein 3	Angja8g005720	Alpha-crystallin A chain	
Angja34413s000010	Stonustoxin subunit alpha	Angja179s000100	---NA---	Angja19g011240	Paired box protein Pax-3; AltName: Full=HuP2	Angja8g015570	Matrix-remodeling-associated protein 5	
Angja2g003390	Beta-mannosidase	Angja1g015640	Alpha-actinin-2	Angja19g014720	Signal peptide peptidase-like 2B	Angja92s000020	Unconventional myosin-XVIIIa	
		Angja21s000230	Bilirubin-inducible fluorescent protein UnaG	Angja19g015300	Tripartite motif-containing protein 35	Angja94s000030	Dynamin-1-like protein	
		Angja2g005980	Collagen and calcium-binding EGF domain-containing protein 1	Angja1g006450	Mitochondrial dicarboxylate carrier	Angja94s000070	Long-chain-fatty-acid--CoA ligase 1	
		Angja2s000030	Sialin; AltName: Full=H(+)/nitrate cotransporter	Angja1g008990	Synaptotagmin-17	Angja9950s000220	Tumor necrosis factor ligand superfamily member 10	
		Angja3g000850	Myogenesis-regulating glycosidase	Angja1g011580	---NA---	Angja99s000220	Centrosome and spindle pole associated protein 1	
		Angja3g005210	Serine/threonine-protein phosphatase with EF-hands 2	Angja1g012150	Gap junction delta-3 protein	Angja9g002250	Lumican; AltName: Full=Keratan sulfate proteoglycan lumican	
		Angja58s000260	60 S ribosomal protein L4-B	Angja1g014050	Mitochondrial import inner membrane translocase subunit Tim23	Angja9g003460	Transmembrane protein 131-like	
		Angja5g013400	Podocan	Angja1g014960	Gamma-adducin	Angja9g004450	Liver carboxylesterase 4	
		Angja6g007070	Cytochrome c oxidase subunit 4 isoform 2, mitochondrial	Angja1g015250	Uroporphyrinogen-III synthase	Angja9g005730	---NA---	
		Angja73s000540	---NA---	Angja1g017860	Transcription factor LBX1	Angja9g006160	Protein phosphatase 1 L	
		Angja77s000150	Neurofilament medium polypeptide	Angja21038s000010	Hepatitis A virus cellular receptor 1 homolog	Angja9g008540	T-complex protein 11-like protein 2	
						Angja9g008620	Nucleoporin Nup37	

**Fig. 4:**
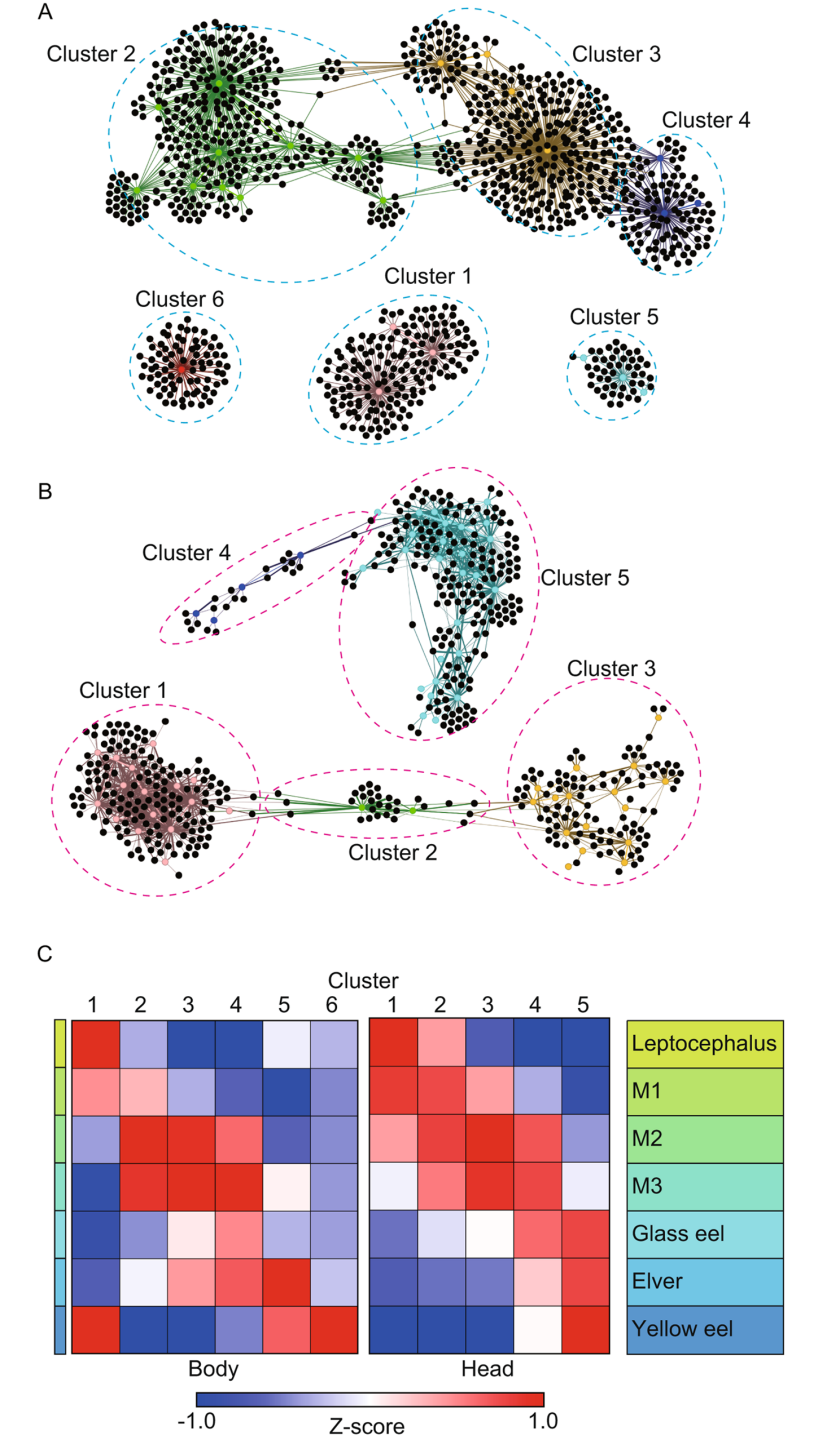
Correlations from network analysis using the most characteristic genes and their co-expression genes. **A**: Body Clusters. **B**: Head Clusters. **C**: Average expression values of each cluster during metamorphosis

Clusters consisting of the most characteristic genes and their correlated genes were constructed. Six clusters in the body and five clusters in the head were constructed (Fig. 4A, B). Clusters 1 to 6 of the body consisted of 165, 377, 376, 133, 49 and 79 genes respectively (Supplementary file 3). Cluster 1 to 5 of the head consisted of 168, 35, 117, 27, and 203 genes, respectively (Supplementary file 4). It has been confirmed that thyroid hormone genes, which are important based on existing knowledge, have been extracted by clusters. Thyroid hormone receptor aA (TRaA) and TRaB belonged to cluster 5 in the body. TRbA belongs to cluster5 in the head. Genes in each cluster differed depending on the developmental stages and GO enrichment analysis revealed the characteristics of the clusters (Fig. 4C). Genes of body cluster 1 were mainly related to intestinal villi, and expression levels of these genes were higher in the leptocephalus stage and the yellow eel stages (Table 2, Supplementary file 5 and 6). Genes of body cluster 2 mainly consisted of proteasome and protein synthesis associated genes that temporarily increased in the M2 and M3 stages. Extracellular matrix and procollagen associated collagen precursors genes were enriched in cluster 3 in the body and these genes increased in the M2 and M3 stage as they were cluster 2. Genes of body cluster 4 mainly consisted of extracellular matrix and lung-associated genes and increased from M2 to Elver stages. The genes of body cluster 5 were related to pore complex and cornification associated genes and increased in the elver and yellow stages. Chloride anion exchanger and polysaccharide digestion-associated genes were enriched in body cluster 6 and these genes increased in the yellow eel stage. Genes of head cluster 1 were higher in the leptocephalus and M1 stages, and mainly consisted of visual, photoreception and cardiac associated genes. Genes of head clusters 2, 3, and 4 continuously fluctuated according to the developmental stage, and extracellular matrix-associated genes were enriched in all of them. In cluster 5, neuron and visual/photoreceptor-associated genes increased, and visual-associated genes were different from those in cluster 1.

**Table 2:** Enriched GO terms of each cluster in the body and head

	Cluster	Enriched GO
Body	1	Intestinal villus association
	2	Proteasome and protein synthesis association
	3	Extracellular matrix, procollagen association
	4	Extracellular matrix and lung association
	5	Pore complex and cornification association
	6	Chloride anion exchanger and polysaccharide digestion
head	1	Visual, Photoreceptive and Cardiac association
	2	extracellular matrix association, (sugar metabolism, cholesterol association)
	3	extracellular matrix and collagen association (ear and roof of mouth association)
	4	Extracellular matrix association
	5	Nerve cell-related, photoreceptor (camera eye retrina) association

## Discussion

Ecological knowledge about the metamorphosis of anguillid eels is limited, primarily because they metamorphose in the ocean and even the large larvae rarely survive net-capture. Although intensive research cruise sampling surveys have succeeded to collect metamorphosing larvae in some cases [24, 64], the metamorphosis of anguillid eels has not been possible to be seen in net captured larvae or directly observed in the oceanic environment. However, the development of the ability to produce Japanese eel seedlings [25] and the recent progression of these techniques [34, 35] allowed us to use metamorphosing larvae in the laboratory for various types of experiments [36–39]. In the present study, we performed RNA-seq analysis on Japanese eel larvae during metamorphosis and on the early juvenile stages. Eels were divided into 7 developmental stages during metamorphosis based on their morphological characteristics, and gene expression data was compared among each stage. Gene expression profiles within the same developmental stage were generally similar in both the body and head. These results suggest that our gene expression data are valid for the analysis of the developmental stages during metamorphosis. The expression profiles of the yellow eel stage in the body were observed to be different than those of the other stages. After offshore and upstream migration, eels settle in rivers or lakes for growth as yellow eels. Previous histological observation revealed that the completion of organogenesis occurs during yellow stages [36]. Our results are quite consistent with those findings. In contrast to the body, apparent differences in expression pattern were not observed in the head between the glass eel, elver, and yellow eel stages. After metamorphosis into glass eels, they enter shallow inland water, which is quite different from the deep ocean environment, and thus their sensory organs need to be prepared to adapt new environments. It is possible that the expression pattern in the head is related to this event. To our knowledge, the RNA-seq dataset of this study is the first stage-series dataset of transcriptome analysis during the larval metamorphosis of anguillid eels.

This analysis enabled us to identify key genes that contribute to the stages of metamorphosis of eels through analysis using the classification model and features provided by the RF algorithm. The commonly used method for comparing expression levels of genes in a dataset is the analysis of differentially expressed genes (DEG) [65, 66]. However, DEG has limitations in its ability to capture non-linear changes that occur in response to dynamic variations of temporal pattens [67]. In this study, we conducted a selective analysis, following the methodology outlined by Asakura et al. (2018) [60] to determine the minimum set of variables necessary for identifying characteristic genes during metamorphosis. This analysis allowed for the identification of genes that are the most characteristic genes that fluctuate nonlinearly during the metamorphosis period. As indicated by the results of the PCA based on the extracted genes, the PCA scores clustered according to developmental stages and expressed a temporal trend. However, when performing selective analysis, adding genes that exhibit the same changes does not contribute to the model accuracy, so they are not included among the “Most characterized genes”. This omission leads to a reduction in the ability to observe the overall phenomenon. Therefore, genes that also increased or decreased along with the “Most characteristic genes” were additionally extracted using correlation coefficients. The clusters of genes obtained through the additional extractions demonstrated temporal variations during the metamorphosis, and by combining them with GO analysis, we were able to extract more refined expression dynamics. The results of this versatile analysis could be a good example of temporal fluctuation of gene expression. Using a correlation coefficient of 0.9, we successfully identified distinct GO features. However, we acknowledge that genes with correlation coefficients below 0.9 might have been overlooked. Hence, it is worth considering the degree of contribution to the GO analysis based on the correlation coefficient in future studies.

To gain further insight into the gene expression dynamics of metamorphosis in anguillid eels, we conducted gene clustering for the most characteristic genes and their correlated genes, and as a result, 6 clusters were constructed for the body and 5 clusters were constructed for the head. Then, the characteristics of the clusters were revealed by GO enrichment analysis. In the body, expression levels of cluster 1 genes were lower during the process of metamorphosis and these genes were mainly related to intestinal villi. During early metamorphosis, the position of the anus moves forward, and the gut shortens. We observed that the eel larvae stopped feeding around the M3 stage. After changing to glass eels, they continued fasting over one month or more in the experimental tank, and while their digestive tract had not completed organogenesis [68], and glass eels are known to not feed when they recruit from open ocean to coastal water [69, 70]. Elvers then migrate upstream during spring to summer and resume feeding. During this time period, food uptake eventually intensifies and then they settled as yellow eels [71]. Our results on the timing of gene expression are consistent with these observation of eels in the natural environment.

Other clusters that were found were related to other biological functions. The GO terms of both cluster 1 and 5 in the head were mainly related to visual function. Genes of cluster 1 were highly expressed at the leptocephalus stage and decreased in expression as the developmental stages progressed, while the genes of cluster 5 had the opposite expression pattern. Anguillid eel larvae are distributed in relatively low-light layers of the upper few hundred meters of the open ocean [22, 72]. In contrast, the habitats of yellow eels are shallow estuarine or inland aquatic areas. Because of this difference in habitat between the larvae and juveniles, retinas of eels change through metamorphosis. The retina of anguillid eel larvae shows a homogenous pattern of rod-like photoreceptors similar to those of deep-sea fish [73, 74], whereas most fish larvae have retina that contain only cone photoreceptors for high-light conditions, with no rods [75]. When metamorphosing from leptocephali into glass eels, the retina of eels change from pure-rod to a duplex retina with rod and cone cells based on morphological analysis [76, 77]. In addition, the phototaxis of eel larvae changes throughout the metamorphosis: they exhibited a clear negative phototaxis in the leptocephalus stage, but no phototaxis was detected after the glass eel stage [78]. The result of the present study clearly reflected these changes.

Genes of many clusters (cluster 3 and 4 in the body, and cluster 2 to 4 in the head) were upregulated during metamorphosis and may be mainly related to the major changes that occur with the extracellular matrix. The body composition of eel larvae was drastically changed in many ways after metamorphosis, but importantly the extracellular matrix contained in a mucinous pouch is converted into new body tissues. Hyaluronan, a polymer of disaccharides composed of glucuronic acid and N-acetylglucosamine, is a main component of the extracellular body matrix of the leptocephalus stage [79, 80]. During metamorphosis, eels convert the hyaluronan that is accumulated during larval stage into other materials such as new tissue like muscle [81, 82]. This unique metabolism of hyaluronan might relate to the association of the extracellular matrix with genes of many clusters. We separated and analyzed cluster 3 and 4 in the body, because genes belonging to cluster 4 exhibited distinct characteristics during the glass eel stage. As expected, the results of the analysis of the changes in these adjacent clusters and GO analysis were similar.

Genes of cluster 2 in the body were also highly expressed in the M1 to M3 stages and were related to proteasome and protein synthesis. We observed that body height shrinks during the M1 to M3 stage, and it is reported that the connective tissues in the dorsal and ventral regions decreased [36]. The proteasome that is a large protein complex responsible for degradation of intracellular proteins, may be associated with these changes.

Genes of cluster 5 in the body related to cornification and these genes peaked in expression at the elver stage. Anguillid eels actively swim upstream or climb over obstructions in rivers during the elver stage [42, 83, 84] and the thickness of the dermis increases [36]. It is speculated that the elevation of cornification related genes is related to skin change to prevent injury during upstream migration. Genes of body cluster 6 were related to the chloride anion exchanger and polysaccharide digestion. These genes were elevated at the yellow eel stage. When they reach the yellow eel stage, organogenesis is complete and the long growth period is started that lasts until the silver eel stage. Eels are euryhaline fish that can spend most of their life as yellow eels in different salinities depending on where they can find suitable habitats: freshwater river, brackish water estuarine, and coastal water saline habitats [85, 86]. In the growth-stage yellow eel phase, they feed on a wide range of invertebrates and fishes [87–95]. Cluster 6 genes may be related to these ecological features of yellow eels. In this analysis, genes that appear to be unrelated to metamorphosis such as lung associated genes and pore complex associated genes were also found to be related. It is not known how these genes are related to eel metamorphosis and we also need to reconsider whether the GO term is appropriate. Further studies may help to more clearly characterize the function of various genes during the metamorphosis of anguillid eels.

## Conclusion

The process of larval metamorphosis of Japanese eels was classified into 7 developmental stages according to their morphological characteristics, and RNA sequencing was used to collect gene expression data from each stage. A total of 354.8 million clean reads from the body and 365.5 million from the head were generated after processing the raw reads. Statistical modeling using the Random Forest algorithm identified the most characteristic genes during the metamorphosis of this species. Using the most characteristic genes and their correlated genes, network analysis, gene clustering and then GO enrichment analysis of the expression patterns and GO terms of each stage were found to be consistent with previous observations and experiments during the larval metamorphosis of anguillid eels.

To our knowledge, this is the first report of transcriptome analysis during the metamorphosis of Japanese eels which are a highly valuable species for aquaculture. The present study contributes substantially to the molecular resources available for this species and will be an important tool for identifying new potential molecular markers for clarifying the mechanisms metamorphosis of anguillid eels.

### Electronic supplementary material

Below is the link to the electronic supplementary material.

Supplementary Material 1

Supplementary Material 2

Supplementary Material 3

Supplementary Material 4

Supplementary Material 5

Supplementary Material 6

Supplementary Material 7

Supplementary Material 8

## Data Availability

All data supporting this research are included in this article and its supplementary file. The raw sequenced reads (accession numbers DRR526476-DRR526530) and normalized RPM data (accession number E-GEAD-673) have been deposited and are links to BioProject accession number PRJDB17339 in the DDBJ BioProject database.

## Abbreviations

TL: Total length
PAL: Preanal length
BD: Body depth
RPM: Reads per million
PCC: Pearson’s correlation coefficient
RF: Random Forest
GO: Gene Ontology
PCA: Principal component analysis
